# Increase and Plateauing of Testicular Cancer Incidence in Austria—A Time Trend Analysis of the Past Four Decades

**DOI:** 10.1016/j.euros.2023.01.005

**Published:** 2023-02-06

**Authors:** Stephan Brönimann, Dong-Ho Mun, Monika Hackl, Lin Yang, Shahrokh F. Shariat, Thomas Waldhoer

**Affiliations:** a Department of Urology, Comprehensive Cancer Center, Medical University of Vienna, Vienna, Austria; b Austrian National Cancer Registry, Statistics Austria, Vienna, Austria; c Department of Cancer Epidemiology and Prevention Research, Alberta Health Services, Calgary, AB, Canada; d Preventive Oncology & Community Health Sciences, Cumming School of Medicine, University of Calgary, Calgary, AB, Canada; e Department of Urology, Weill Cornell Medical College, New York, NY, USA; f Department of Urology, University of Texas Southwestern, Dallas, TX, USA; g Department of Urology, Second Faculty of Medicine, Charles University, Prague, Czech Republic; h Hourani Center for Applied Scientific Research, Al-Ahliyya Amman University, Amman, Jordan; i Karl Landsteiner Institute, Vienna, Austria; j Centre for Public Health, Department of Epidemiology, Medical University of Vienna, Vienna, Austria

**Keywords:** Testicular cancer, Incidence, Austria, Testicular germ cell tumor, Seminoma, Nonseminoma

## Abstract

**Background:**

Testicular germ cell tumors (TGCTs) are the most common malignant tumors in young men. Despite considerable geographic, ethnic, and temporal variations in the incidence of TGCTs, without convincing explanation, incidence rates of TGCTs have been increasing in many countries since, at least, the mid-20th century.

**Objective:**

To investigate the incidence rates of TGCTs in Austria by analyzing data from the Austrian Cancer Registry.

**Design, setting, and participants:**

Available data between 1983 and 2018 were provided by the Austrian National Cancer Registry and analyzed retrospectively.

**Outcome measurements and statistical analysis:**

Germ cell tumors derived from germ cell neoplasia in situ were classified into seminomas and nonseminomas. Age-specific incidence rates and age-standardized rates were calculated. Annual percent changes (APCs) and average annual percent changes in incidence rates were determined to describe trends from 1983 to 2018. All statistical analyses were performed using SAS version 9.4 and joinpoint.

**Results and limitations:**

The study population consists of 11 705 patients diagnosed with TGCTs. The median age at diagnosis was 37.7 yr. The standardized incidence rate of TGCTs increased significantly (*p* < 0.0001) from 4.1 (3.4, 4.8) per 100 000 in 1983 to 8.7 (7.9, 9.6) per 100 000 in 2018 by an average APC of 1.74 (1.20, 2.29). The joinpoint regression revealed a change point in time trend in 1995 with an APC of 4.24 (2.77, 5.72) before 1995 and an APC of 0.47 (0.06, 0.89) thereafter. Incidence rates were about twice as high for seminomas as for nonseminomas. A trend analysis by age group showed that the highest TGCT incidence rate was observed among men aged 30–40 yr, with a steep increase before 1995.

**Conclusions:**

The incidence rate of TGCTs increased in Austria over the past decades and appears to have reached a plateau at a high level. A time trend analysis by age group for the overall incidence was highest in men aged 30–40 yr, with a steep increase before 1995. These data should lead to awareness campaigns and research to further investigate the causes of this development.

**Patient summary:**

We reviewed the data between 1983 and 2018 provided by the Austrian National Cancer Registry to analyze the incidence and incidence trend in testicular cancer. Testicular cancer shows an increasing incidence in Austria. The overall incidence was highest in men aged 30–40 yr, with a steep increase before 1995. The incidence appears to have reached a plateau at a high level in recent years.

## Take Home Message

The incidence rate of testicular germ cell tumors (TGCTs) increased in Austria over the past decades and appears to have reached a plateau at a high level. The median age at diagnosis was 37.7 yr overall, 38.2 yr for patients with seminomas, and 30.3 yr for patients with nonseminomas. The standardized incidence rate of TGCTs increased significantly by an average annual percent change of 1.74 (1.20, 2.29). The joinpoint regression revealed a trend change point in 1995. A trend analysis by age group showed that the highest TGCT incidence rate was observed among men aged 30–40 yr, with a steep increase before 1995.

## 1 Introduction

Testicular cancer is the most common malignant tumor in young men, with testicular germ cell tumors (TGCTs) representing the vast majority (98%) [1], [2]. Worldwide, there are considerable geographic, ethnic, and temporal variations in the incidence of TGCTs, with the highest incidence rates being reported in Scandinavian countries [3]. Indeed, all the top 12 highest incidence countries are located in Northern, Central, Southern, or Eastern Europe, with Norway (age-standardized rate: 11.5 cases per 100 000 person-years) and Denmark (10.2 per 100 000 person-years) followed by Switzerland (8.9 per 100 000 person-years) showing the highest incidence rates. The lowest incidence of TGCTs is found in Africa (Uganda 0.3 per 100 000 person-years) and Asia (Thailand 0.5 per 100 000 person-years, India 0.6 per 100 000 person-years, Philippines 0.8 per 100 000 person-years, and China 1.5 per 100 000 person-years) [4].

Interestingly, without convincing explanation, incidence rates of TGCTs have been increasing in many countries since, at least, the mid-20th century. To date, no study reported data on trends in the incidence rates of TGCTs and their subtypes (seminomas and nonseminomas) in Austria; we aimed to investigate the incidence rates of TGCTs in Austria by analyzing data from the Austrian Cancer Registry.

## 2 Patients and methods

Available data between 1983 and 2018 were provided by the Austrian National Cancer Registry (ANCR). The data set included histology of the tumor entity according to the International Classification of Diseases for Oncology (ICD-O-3, 3rd edition), date of diagnosis, and age of diagnosis. We subclassified the germ cell tumors derived from germ cell neoplasia in situ based on the 2016 update of the World Health Organization pathological classification into seminomas and nonseminomas. Seminomas included seminomas and anaplastic seminomas, while nonseminomas included embryonal carcinoma, yolk-sac tumor, teratoma, choriocarcinoma, and those classified simply as nonseminomatous germ cell tumors and germinal mixed tumors [2].

### 2.1 Statistical analyses

Age-specific incidence rates were calculated in 3-yr cycles to obtain a stable estimation. Age-standardized rates were calculated by year using the European standard population [5].

Annual percent changes (APCs) as well as average APCs in incidence rates were calculated to describe trends from 1983 to 2018. Age was grouped as 0–<20, 20–<30, 30–<40, …, 80+ for age-specific analyses. In addition, APCs were described by histology and age group to identify trends by subgroups. All statistical analyses were performed using SAS version 9.4 (SAS Institute, Cary NC, USA) and joinpoint (Joinpoint Regression Program, version 4.7.0.0, Statistical Research and Applications Branch, National Cancer Institute, Bethesda, MD, USA). For a joinpoint analysis of trends based on yearly rates, the maximum number of joinpoints as well as the minimum number of either point was set to 2, and the minimum number between joinpoints was set to 3. We used a permutation test, set calculation of confidence intervals (CIs) to parametric, and used an uncorrelated error model. Variability of rates and APCs were described by 95% CIs and presented in brackets. We considered *p* values <0.05 to be statistically significant. No adjustment for multiple testing was done; accordingly, *p* values are to be interpreted explanatorily only. This study does not require approval by an Institutional Review Board because ANCR collects data on a legal basis (Cancer Statistics Act 1969 and Cancer Statistics Ordinance 2019) and provides it to researchers in anonymized form only.

## 3 Results

The study population consists of 11 705 patients diagnosed with testicular cancer between 1983 and 2018 in Austria. Owing to a lack of subclassification from 1983 to 1989, data on the differentiation between seminomas and nonseminomas are missing up to 1989 and therefore both groups are shown in total only. From 1990, 10 170 patients were diagnosed with testicular carcinoma, of whom 5935 (58.3%) were diagnosed with a seminoma and 3224 (31.7%) with a nonseminoma.

The median age at diagnosis was 37.7 yr overall, 38.2 yr for patients with seminomas, and 30.3 yr for patients with nonseminomas.

**Fig. 1:**
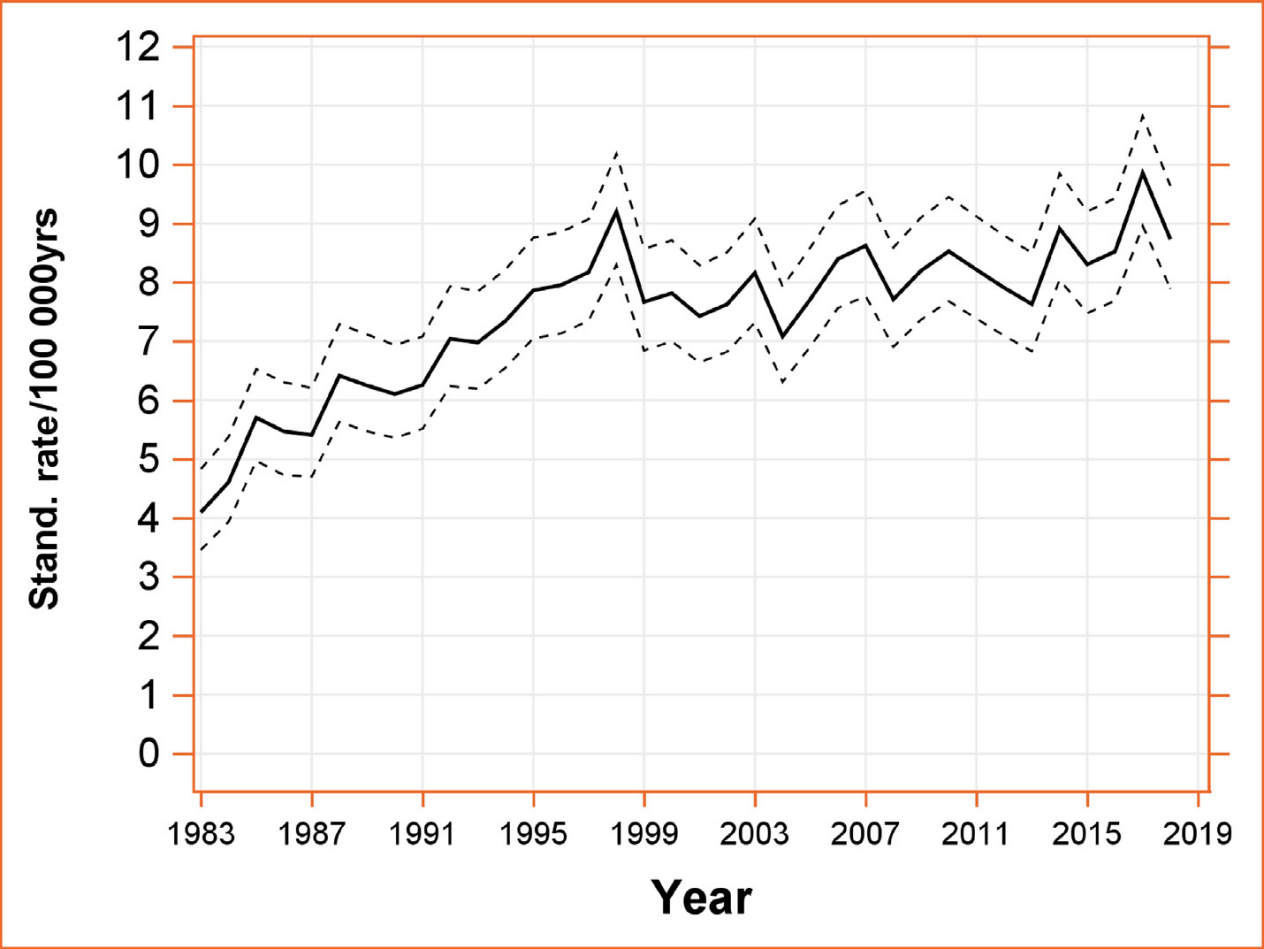
Standardized incidence rate of TGCTs in Austria from 1983–2018. Stand. = standardized; TGCT = testicular germ cell tumor.

The standardized incidence rate of TGCTs increased significantly (*p* < 0.0001) from 4.1 (3.4, 4.8) per 100 000 in 1983 to 8.7 (7.9, 9.6) per 100 000 in 2018 (Fig. 1) by an APC of 1.74 (1.20, 2.29). The joinpoint regression revealed a change point in time trend in 1995 with an APC of 4.24 (2.77, 5.72) before 1995 and an APC of 0.47 (0.06, 0.89) thereafter.

**Fig. 2:**
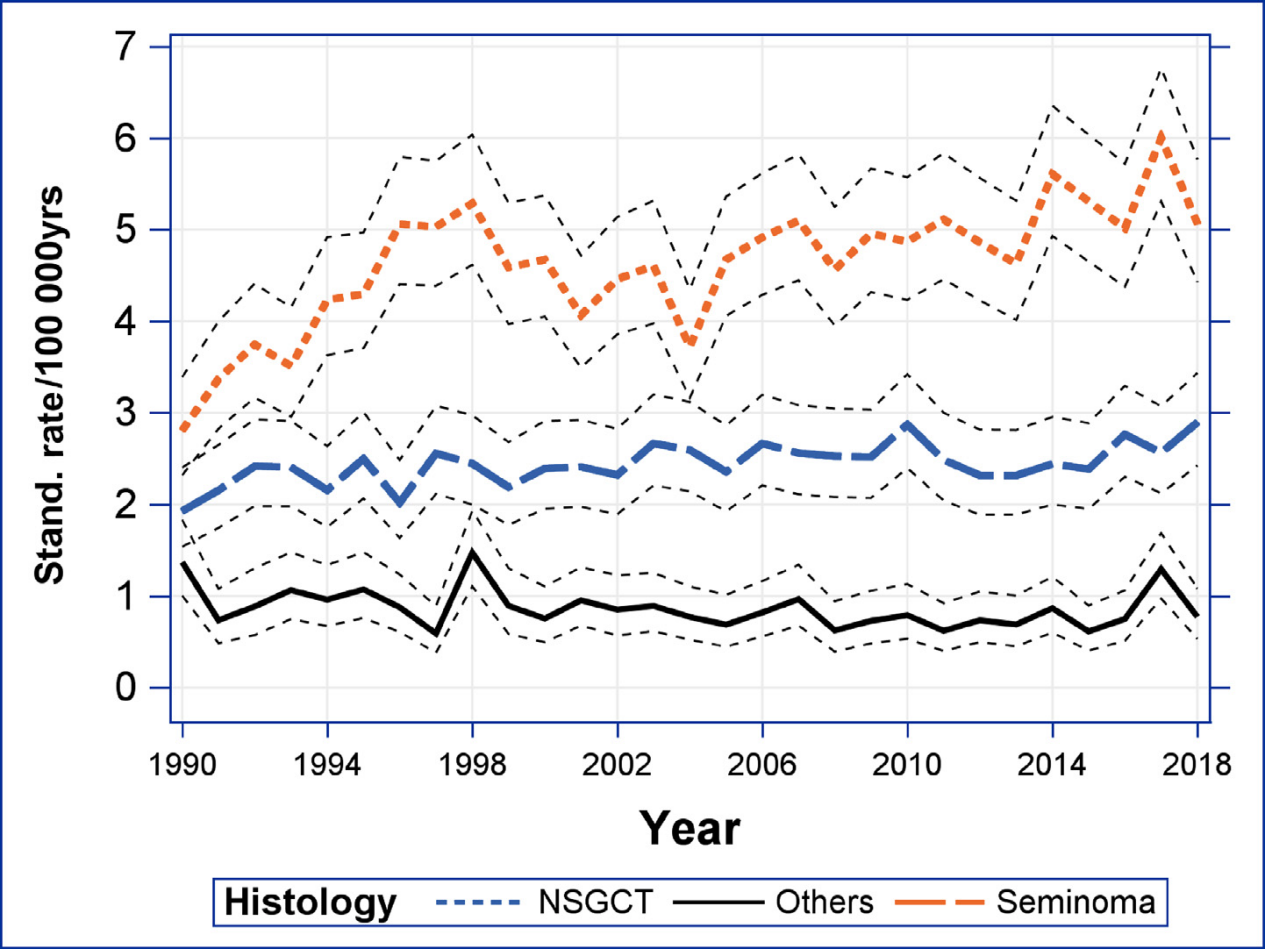
Standardized incidence rates of TGCTs in Austria from 1983 to 2018 by histological subtype. NSGCT = nonseminoma germ cell tumor; Stand. = standardized; TGCT = testicular germ cell tumor.

Incidence rates were about twice as high for seminomas as for nonseminomas from 1990 to 2018 (see Fig. 2). The standardized incidence of seminomas increased from 2.8 (2.3, 3.4) per 100 000 in 1990 to 5.1 (4.4, 5.8) per 100 000 in 2018. The joinpoint regression revealed a significant (*p* < 0.05) joinpoint at 1996 with an APC of 7.06 (1.87, 12.51) before 1996 and an APC of 0.59 (0.02, 1.16) thereafter. The APC between 1990 and 2018 was 1.94 (0.83, 3.07).

The incidence of nonseminomas increased from 1.9 (1.6, 2.4) per 100 000 in 1990 to 2.9 (2.4, 3.4) per 100 000 in 2018. During this time period, the annual percent change was 0.63 (0.28, 0.98) for this group with no significant change point.

A trend analysis by age group showed that the highest TGCT incidence rate was observed among men aged 30–40 yr, with a steep increase before 1995 (Supplementary Fig. 1). A similar steep increase but for the whole time period was observed in age groups 40–50 and 50–60 yr.

A trend analysis by age group for seminomas showed a similar pattern to TGCTs except for a lower incidence rate observed among men aged 20–30 yr than in those aged 40–50 yr (Supplementary Fig. 2).

For nonseminomas, the highest incidence rate was observed among men aged 20–30 yr (see Supplementary Fig. 3). For both seminomas and nonseminomas, the absolute increase in incidence rate was most prominent in the subgroup of men aged 40–50 years (see APC by age in Supplementary Table 1).

## 4 Discussion

This study is the first to investigate the incidence rates of TGCTs in Austria. Our analysis showed that men diagnosed with a seminoma were approximately 8 yr older than those diagnosed with a nonseminoma. This is consistent with the literature, which reports an age difference of 6–9 yr between seminomas and nonseminomas [6], [7], [8].

From 1990 through 2018, seminoma diagnoses were approximately twice that of nonseminoma diagnoses (58.3% vs 31.7%). Similar to the present findings, previous studies have also demonstrated a generally higher incidence of seminomas than of nonseminomas [4]. The highest proportion of seminomas was reported in Germany (65%), Italy (62%), and Switzerland (61%) [9], [10], [11].

Regarding the time trend, the standardized incidence rate for TGCTs increased significantly, with an average APC of 1.74 between 1983 and 2018. The joinpoint regression revealed a trend change point in 1995. Considering the temporal trend in other countries, numerous studies have been published, with the general consensus that the absolute number of TGCTs is rising. As a result of the demographic effects of the aging population, the incidence of most tumors is increasing. As TGCTs primarily affect young men, a population group that is diminishing in numbers in most European countries, one would anticipate a reduction in TGCT diagnoses. However, the rapidly rising incidence rates observed offset the demographic development. According to an analysis of 28 European countries, the absolute number of TGCT diagnoses is predicted to increase by 13% by 2035 [12]. Especially countries with a relatively low TGCT incidence experienced high growth rates, ranging between 3% and 5% annually. However, as shown in our analyses, we demonstrated a change point in time trend in 1995; the rate of increase is slowing down in recent years in some population subgroups. This slowed down rising trend is especially described in high-incidence countries, such as Norway (2.4% [95% CI 2.0–2.8]) and Denmark (0.8% [95% CI 0.4–1.3]), with APCs nearing 0% in these countries in more recent decades [4]. Additionally, Chia et al [13] reported plateauing incidence rates in England and Switzerland. According to Shah et al [14], incidence rates among Asian/Pacific Islander and American Indian/Alaska Native seem to stabilize as well, while the rising incidence rates in previously European lower-incidence countries, such as Croatia and Slovakia, appear to have accelerated in recent decades, narrowing the gap among European countries [4]. Concluding, the incidence rate in high-incidence countries, such as Norway, Denmark, Germany, Switzerland, and England, showed stabilization, potentially indicating the beginning of a plateau phase after a peak incidence having possibly been reached [13], [15], which is also in concordance with our observation for Austria. On the contrary, the majority of countries with relatively low TGCT incidence rates still report a 3–5% average annual growth in incidence rates [4].

When examining the standardized incidence rates of seminomas and nonseminomas separately, we were able to show almost doubling of the incidence rate of seminomas from 1990 to 2018. However, there was a significant change point in 1996, with rapidly declining APCs thereafter. The incidence of nonseminomas increased as well, but less markedly and without a clear change point. In addition, existing literature indicates that the number of seminomas is increasing more rapidly than that of nonseminomas [4].

Taking a closer look at the affected age groups, our time trend analysis for all diagnoses showed that the highest increase in incidence rate was observed among men aged 30–40 yr, with a steep increase before the year 1995. A similar steep increase from 1990 through 2018 was observed in age groups 40–50 and 50–60 yr. In seminomas as well as nonseminomas, the increase in the incidence rate was most prominent in the age group of men aged 40–50 yr (see APCs by age in Supplementary Table 1).

In the Austrian population, this development led to an incidence rate of 8.7 (7.9, 9.6) per 100 000 men in 2018. Regarding the general incidence rate worldwide, the reported geographic, ethnic, and temporal variations are considerable. Particularly high incidences were reported in studies from Denmark (11.5 per 100 000 men around 1995), Norway (9.1 per 100 000 men), and Switzerland (11.0 per 100 000 men in Vaud), whereas Japan, Finland, and Israel reported the lowest incidence [16]. It has been suggested that approximately 25% of TGCT susceptibility is due to genetic effects [17]. Additional evidence in favor of a role for inherited susceptibility comes from familial studies that have found that brothers have a five- to 19-fold higher risk and sons a two- to fourfold increased risk compared with men with no affected relatives [18], [19], [20]. Therefore, differences in the genetic background might explain a proportion of the differences in incidence rates. Even so, incidence rates of TGCTs differ also among geographically and culturally close countries, as it is approximately twofold higher in Norway than in Sweden, without any genetic differences being found between the general populations living in these countries [21]. This fact supports the hypothesis that environment plays a key role, which is further supported by migration studies [22], [23]. Furthermore, the steadily increasing incidence rate in TGCTs, which is reported since the mid-20th century, cannot be explained fully by genetic susceptibility and supports the existence of exogenous risk factors [24], [25], [26]. Considering this aspect, hormones and endocrine disruptors have been suspected to influence tumor genesis. There is, for example, evidence suggesting that exposure to dichlorodiphenyltrichloroethylene (p,p′-DDE), a pesticide used up to the 1980s, can potentially act as a hormonal disruptor, which has been linked to TGCT genesis [26].

The increasing incidence rate in TGCTs observed in the present study is unlikely a mere artifact of changes in diagnostics, classification, or cancer registration practices. TGCTs are histologically well characterized; hence, cross-classification with other cancer types is not expected. Furthermore, if left undiagnosed and untreated, germ cell tumors have a fatal natural history and will eventually be detected postmortem. In addition, changes in population screening practices cannot explain the trend, because no testicular cancer screening programs exist in any country. The theory that competing morbidity may alter the incidence rate is also unlikely because there are few competing risks among young men. Additionally, migrational trends are unlikely to lead to this development as most migration is from low- to high-incidence areas.

This study is based on data from the Austrian Cancer Registry, to which reporting cancer diagnoses are mandatory; hence, it represents nationwide cancer cases [27].

Finally, as progress of TGCTs appears under the influence of sexual hormones, which results in a long time lag between exposure and TGCT occurrence, identification of the risk factors responsible for the steep increase of the incidence rate remains challenging. Although we were able to show the time trend of TGCTs in Austria, the reason for the increase of the incidence and high plateauing in TGCTs remains unknown, which necessitates further investigations for this observation and underlying risk factors.

## 5 Conclusions

Our analyses showed that the incidence rate of TGCTs increased in Austria over the past decades and appears to have reached a plateau at a high level. The time trend analysis by age group for the overall incidence was highest in men aged 30–40 yr, with a steep increase before 1995. For both seminomas and nonseminomas, the increase in incidence rate was most prominent among men aged 40–50 yr. These data should lead to awareness campaigns and research to further investigate the causes of this development. When examining the standardized incidence rates of seminomas and nonseminomas separately, we were able to show almost doubling of the incidence rate of seminomas from 1990 to 2018. However, there was a significant change point in 1996 with rapidly declining APCs thereafter. The incidence of nonseminomas increased as well, but less markedly and without a clear change point.

  ***Author contributions*:** Stephan Brönimann had full access to all the data in the study and takes responsibility for the integrity of the data and the accuracy of the data analysis.

  *Study concept and design*: Brönimann, Ho Mun.

*Acquisition of data*: Waldhoer, Hackl.

*Analysis and interpretation of data*: Brönimann, Waldhoer.

*Drafting of the manuscript*: Brönimann, Ho Mun.

*Critical revision of the manuscript for important intellectual content*: Hackl, Yang, Shariat.

*Statistical analysis*: Waldhoer.

*Obtaining funding*: None.

*Administrative, technical, or material support*: Waldhoer.

*Supervision*: Shariat, Yang.

*Other*: None.

  ***Financial disclosures:*** Stephan Brönimann certifies that all conflicts of interest, including specific financial interests and relationships and affiliations relevant to the subject matter or materials discussed in the manuscript (eg, employment/affiliation, grants or funding, consultancies, honoraria, stock ownership or options, expert testimony, royalties, or patents filed, received, or pending), are the following: None.

  ***Funding/Support and role of the sponsor*:** None.
