# Supplementary material for: Increase and Plateauing of Testicular Cancer Incidence in Austria—A Time Trend Analysis of the Past Four Decades
Source: Eur Urol Open Sci. 2023 Feb 6;49:104–9. doi: 10.1016/j.euros.2023.01.005 (PMC9974997; doi:10.1016/j.euros.2023.01.005)
Supplement: Supplementary data 1 [file mmc1.docx]

*
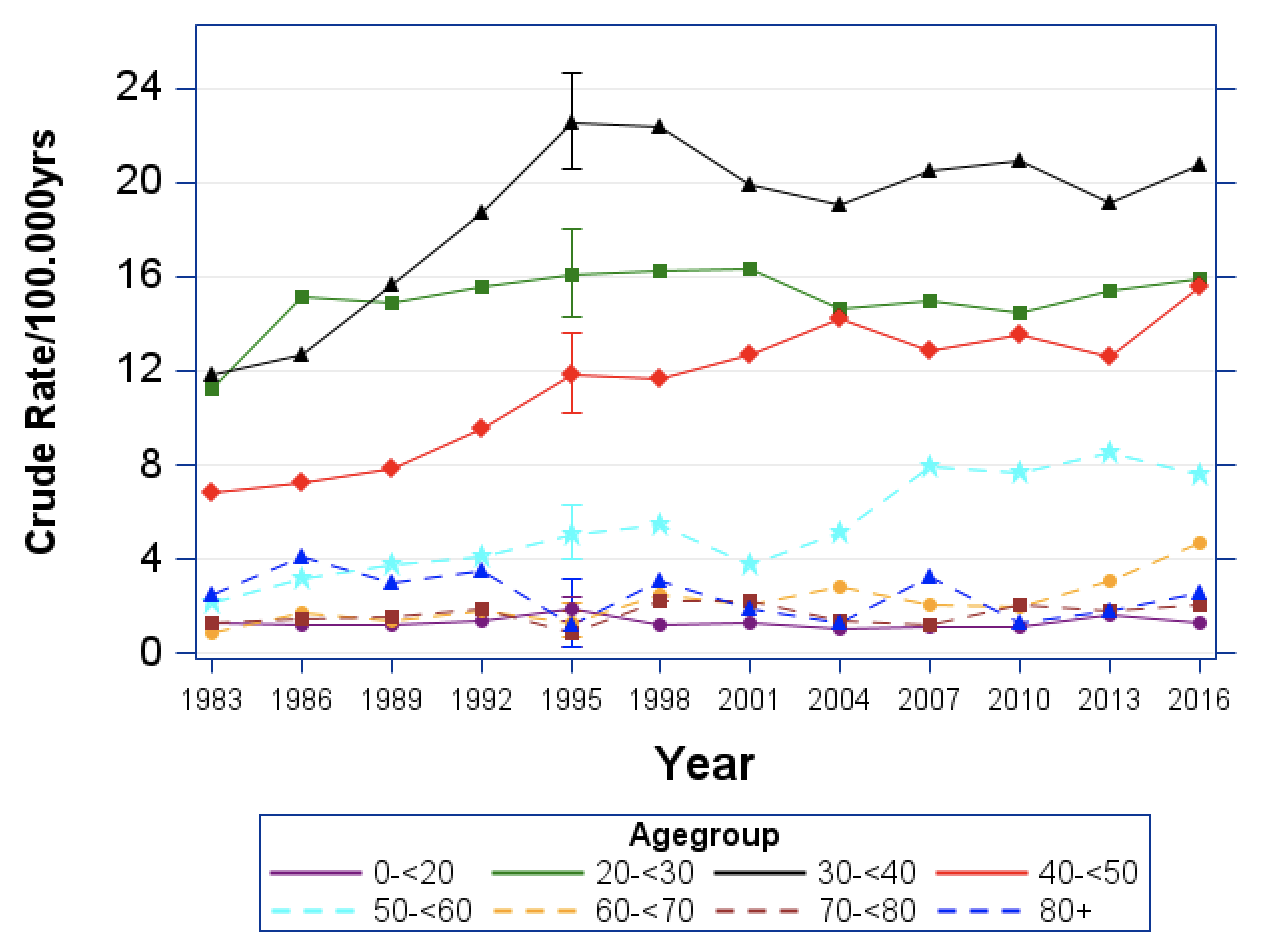
*

*Supplementary Figure 1: Age-specific rate of TGCT by 3-year cycles from 1983-2018*

*
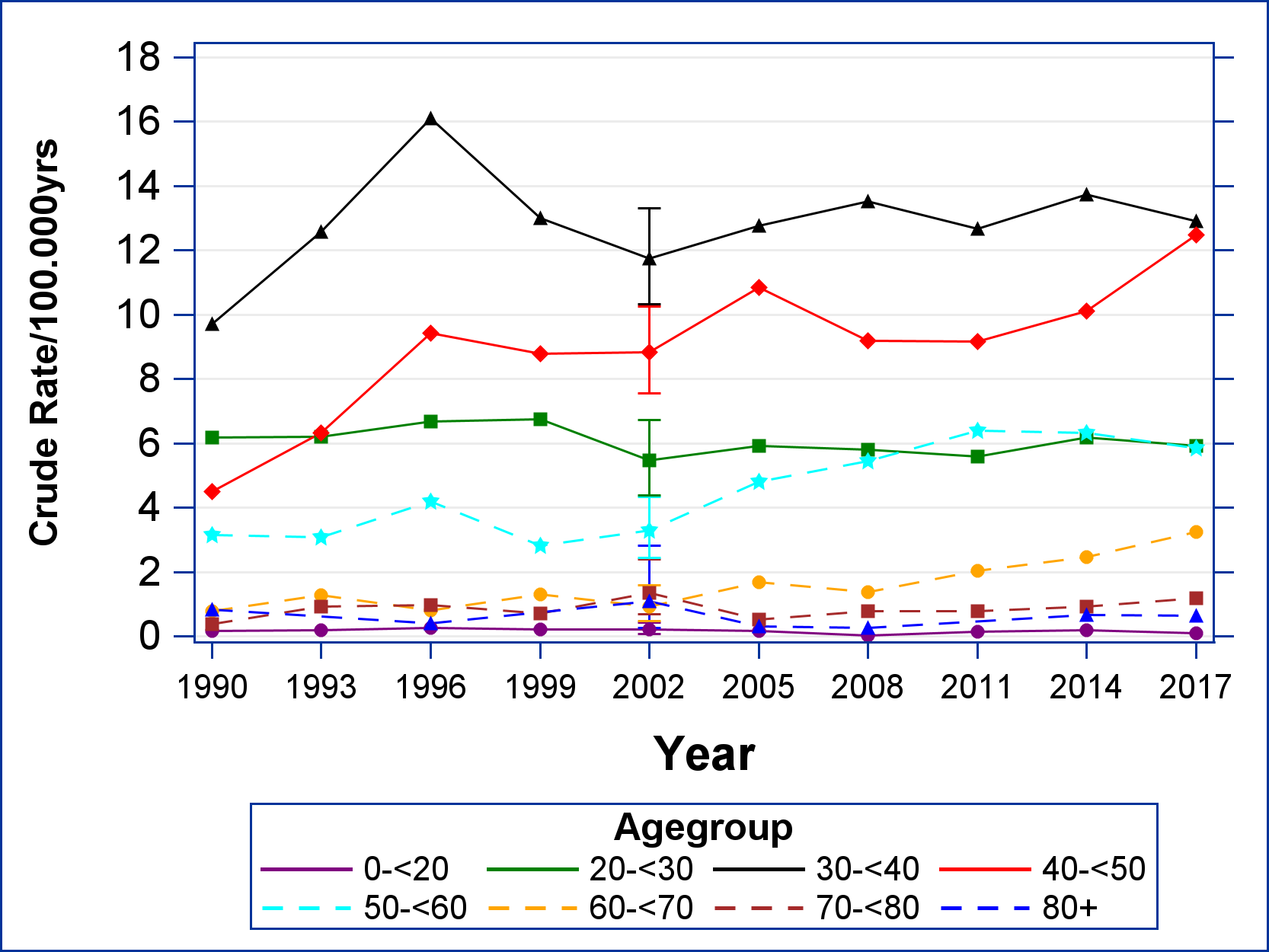
*

*Supplementary Figure 2: Age-specific rate of seminoma by 3-year cycles from 1990-2018*

*Supplementary Figure 3: Age specific rate of non-seminoma
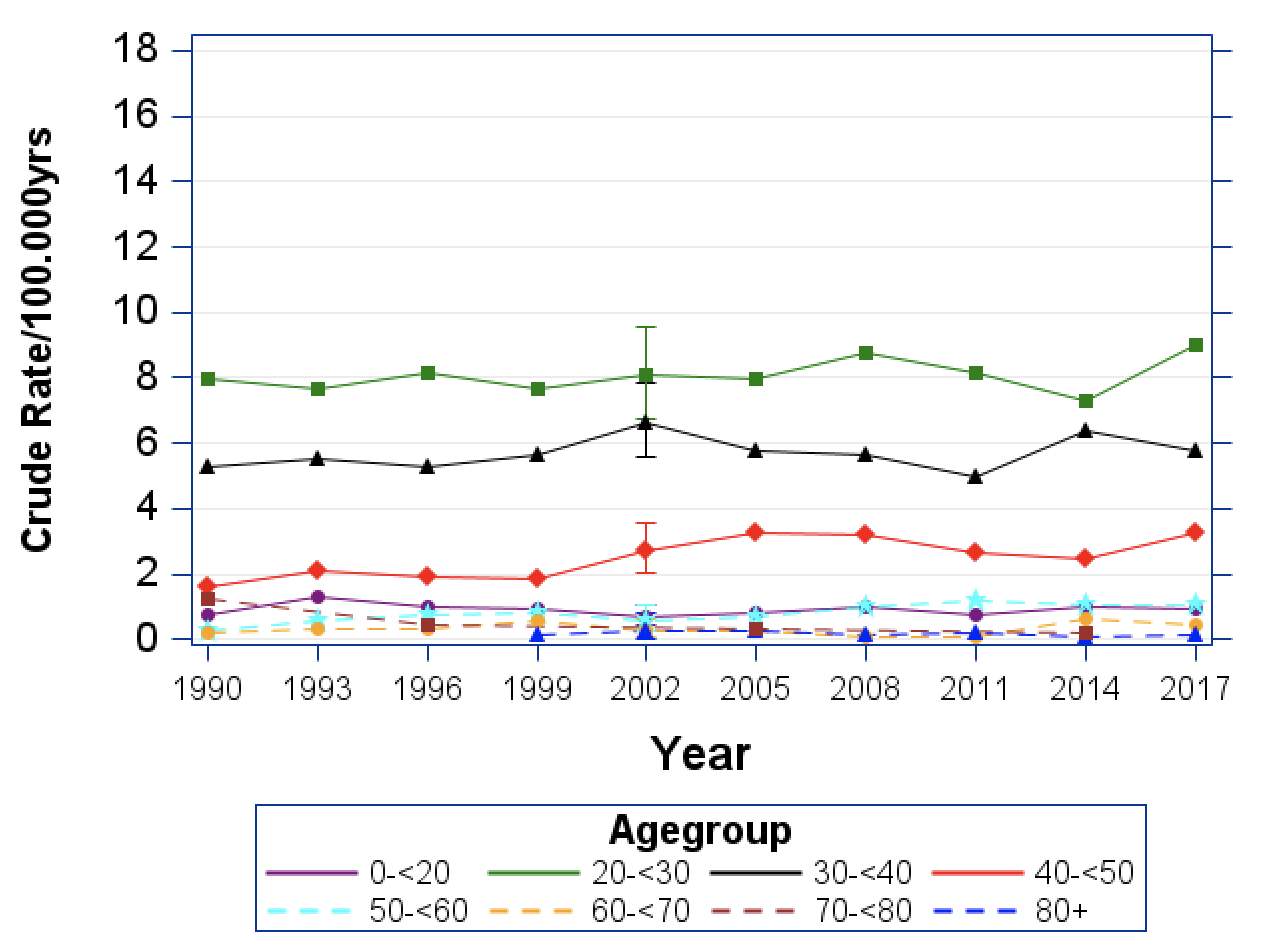
 by 3-year cycles from 1990-2018*
