# Supplementary material for: Increase and Plateauing of Testicular Cancer Incidence in Austria—A Time Trend Analysis of the Past Four Decades
Source: Eur Urol Open Sci. 2023 Feb 6;49:104–9. doi: 10.1016/j.euros.2023.01.005 (PMC9974997; doi:10.1016/j.euros.2023.01.005)
Supplement: Supplementary data 2 [file mmc2.docx]

|  | Whole cohort |  |  |  | Seminoma | |  |  | Non-seminoma | |  |
| --- | --- | --- | --- | --- | --- | --- | --- | --- | --- | --- | --- |
| Age group | APC | Lower CL | Upper CL |  | APC | Lower CL | UpperC L |  | APC | Lower CL | Upper CL |
| 0-<20 | -0,17 | -0,98 | 0,64 |  | -1,71 | -4,53 | 1,18 |  | -0,42 | -2,11 | 1,29 |
| 20-<30 | 0,29 | -0,29 | 0,87 |  | -0,34 | -0,88 | 0,21 |  | 0,21 | -0,30 | 0,72 |
| 30-<40 | 0,97 | -0,08 | 2,03 |  | 0,24 | -0,95 | 1,45 |  | 0,33 | -0,47 | 1,13 |
| 40-<50 | 2,20 | 1,22 | 3,19 |  | 2,04 | 0,52 | 3,57 |  | 2,05 | 0,35 | 3,79 |
| 50-<60 | 3,23 | 2,10 | 4,38 |  | 3,13 | 1,72 | 4,55 |  | 3,13 | 1,00 | 5,31 |
| 60-<70 | 3,67 | 2,21 | 5,16 |  | 4,85 | 2,92 | 6,82 |  | 1,92 | -2,86 | 6,93 |
| 70-<80 | 1,19 | 0,06 | 2,33 |  | 0,81 | -2,04 | 3,75 |  | -2,75 | -7,46 | 2,21 |
| 80+ | -1,68 | -3,87 | 0,55 |  | -0,87 | -4,60 | 3,00 |  | -7,14 | -9,85 | -4,35 |

Supplementary Table 1: Annual percentage change (APC) by age group in the whole cohort, in patients with seminoma and non-seminoma
